# Influence of luting space settings and polymerization-induced cement shrinkage and deformation/adaptation of CAD/CAM crowns

**DOI:** 10.1590/1807-3107bor-2025.vol39.074

**Published:** 2025-07-07

**Authors:** Leandro Maruki PEREIRA, Bárbara Inácio de MELO, Aline Aredes BICALHO, Rayssa Rodrigues PEREIRA, Marcel Santana PRUDENTE, Carlos José SOARES, Flávio Domingues das NEVES

**Affiliations:** (a) Universidade Federal de Uberlândia – UFU, School of Dentistry, Department of Occlusion, Fixed Prosthodontics and Dental Materials, Uberlândia, MG, Brazil.; (b) Universidade Federal de Uberlândia – UFU, Health Technical School (ESTES), Department of Basic, Technical and Technological Education, Uberlândia, MG, Brazil.; (c) Universidade Federal de Uberlândia – UFU, School of Dentistry, Department of Dentistry and Dental Materials, Uberlândia, MG, Brazil.

**Keywords:** Crowns, Dental Marginal Adaptation, Cementation

## Abstract

This study aimed to evaluate the adaptation of CAD/CAM crowns and the impact of resin cement polymerization contraction on crown deformation under different cementation configurations. Thirty human molars were randomly divided into three groups (n = 10) to manufacture ceramic crowns with three different cementation space configurations: LD40 (40 μm), LD80 (80 μm), and LD160 (160 μm). The samples were subjected to computerized microtomography scanning for measuring internal and marginal adaptation, and internal fit was analyzed at occlusal, axial, axiogingival points. Five light-curing protocols were used to verify the wavelength spectrum peak of the curing device and the irradiance delivered using two situations: with 0 mm sensor distance and through a ceramic of differing thickness, and an external crown deformation (ECD) test. The highest statistically significant vertical fit was obtained for group LD40, independent of region. LD160 yielded the highest ECD values, independent of positioning of the buccal and distal strain gauge. The highest internal fit was observed at occlusal and axial points of the LD160 group (P<0.001), and LD80 yielded the lowest values at the axiogingival point (p = 0.003, p = 0.006). The irradiance delivered was reduced significantly upon interposition of the ceramic block to the sensor. The peak wavelength for curing was similar among the LD groups. The luting space configurations significantly influenced vertical fit and the ECD results for the CAD/CAM ceramics owing to polymerization shrinkage. Finally, variations in ceramic thickness at different sites affected both the irradiance by the curing device and ECD results.

## Introduction

CAD/CAM systems and their software could be standardizing luting space because this parameter could be accurately set using system software.^1-9^ The luting space of ceramic crowns is filled with resin cement that has a lower elastic modulus than the ceramic and, normally, an elastic modulus similar to that present in dentin, thereby preventing fracture of the crown during chewing.^10,11^ Considering that ceramics are inherently brittle, choosing a reinforced ceramic for crowns, an appropriate luting space, and resin cement are vital for the success of the restoration.^2,5,6,11^ However, a smaller inner space may increase the difficulty of setting the crown, which may result in a misfit of the vertical margin.^20,21^ In contrast, a larger internal space generates a thicker layer of resin cement, which generates stress within both the restorative cavities and material and can lead to bonding failure.^3,4^

Different settings for luting space have been reported for CAD/CAM ceramic crowns fabricated by the CEREC system.^12^ They range from 0 to 50 μm, resulting in crowns with a marginal fit between 36.8 and 164.0 μm and internal fit between 109.5 and 162.0 μm.^2,9,9,13-16^ No reliable reproduction^2,7,12-14,17,18^ of the selected setting of luting space has been observed after measuring internal and marginal fit. In addition, an in vitro test showed that the ideal thickness of cement should range from 25 to 40 μm,^19^ although this range is rarely achieved clinically.

Throughout the cement polymerization process, the material undergoes contraction.^20^ The shrinkage resulting from this process is directly linked to the deformation of ceramic materials and the fit of the crown adaptation.^21,22^ The stress generated by polymerization shrinkage depends on multiple factors, such as curing-light intensity, photoactivation time, elapsed time between crown cementation and light activation, elastic modulus of the resin cement, and the amount of tooth structure remaining.^21,23-25^ A larger internal space accommodates a greater amount of resin cement, and this may contribute to deformation of the adjacent structure, which can result in an increased transfer of shrinkage stress to the surface of a ceramic crown.^3,4^ Therefore, testing the effect of internal space size can be used to verify the influence of the shrinkage process on ceramic crowns.

During polymerization, it is important to analyze the degree of conversion,^26-28^ which is influenced by irradiance, or the intensity of the light source used to cure the cementing agent, which affects the cementation process.^29,30^ If there is sufficient light to ensure complete polymerization, the subsequent effect is to produce a polymer network, and polymerization shrinkage is a natural consequence.^25,29,31^ The type of ceramic used and its thickness could lead to some attenuation and affect the degree of conversion of resin cement polymerization.^30,32^ Inadequate polymerization, characterized by a low degree of conversion, decreases the mechanical properties of the cement and increases its water absorption and solubility, and the increased amount of residual monomers may cause pulp irritation and irreversible pulpitis and thus decrease the bonding resin cements.^30,33^

Polymerization shrinkage is influenced by irradiance,^34-36^ which may affect the adaptation of CAD/CAM crowns depending on the luting space settings, aspects that are not conclusively evidenced in the literature. Thus, the objective this study was to analyze the luting space settings of CAD/CAM disilicate ceramic crowns generated by using the intraoral camera of the CEREC Omnicam with software version 4.2.5 system with the following considerations: (a) marginal and internal fit was assessed by μ-CT analyses, (b) crown deformation during polymerization shrinkage of cement was assessed by the strain gauge method, and (c) the amount of irradiance and light spectrum of the curing light unit through the ceramic was measured. The null hypothesis was that the luting space settings do not influence internal fit, marginal fit, or ceramic crown deformation resulting from polymerization shrinkage of the resin cement and that there would be no attenuation of irradiance. The other null hypothesis was that no attenuation of irradiance or light spectrum of the curing light unit through the ceramic.

## Methods

### Selection and preparation of teeth

**Figure 1:**
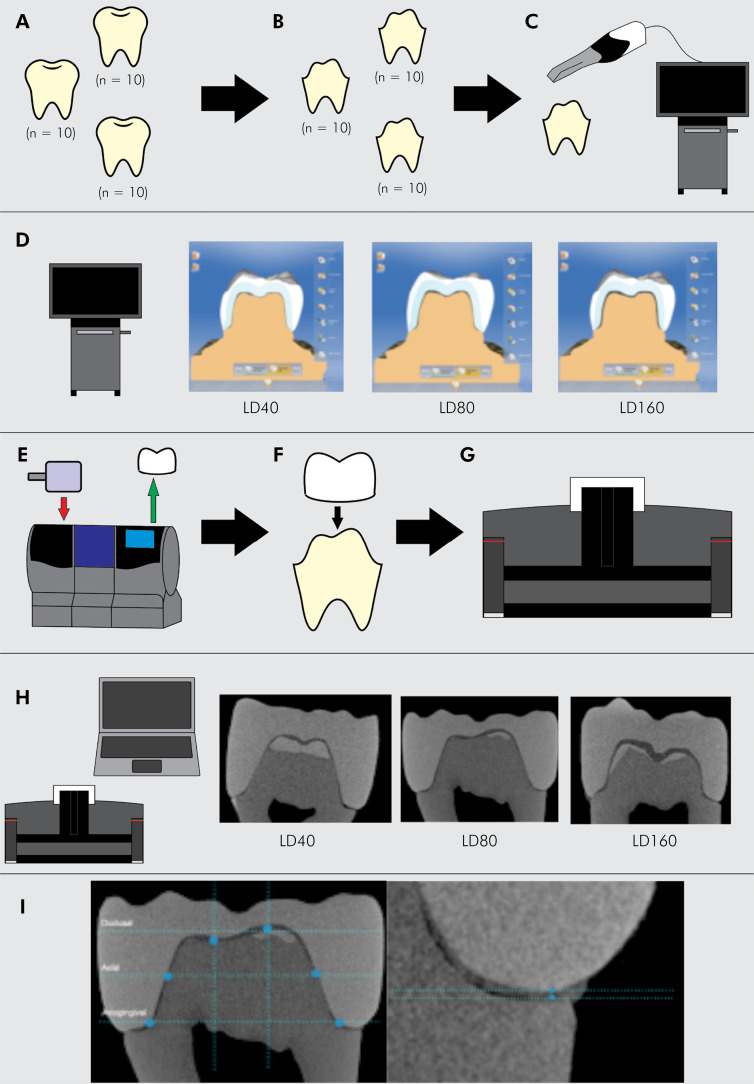
Experimental methodology. (A) Tooth selection, (B) tooth preparation, (C) scanning process, (D) crown design, (E) crown fabrication, (F) fixation process, (G) micro-computed tomography, (H) reconstruction, and (I) internal and marginal measurement procedures.

**Figure 2:**
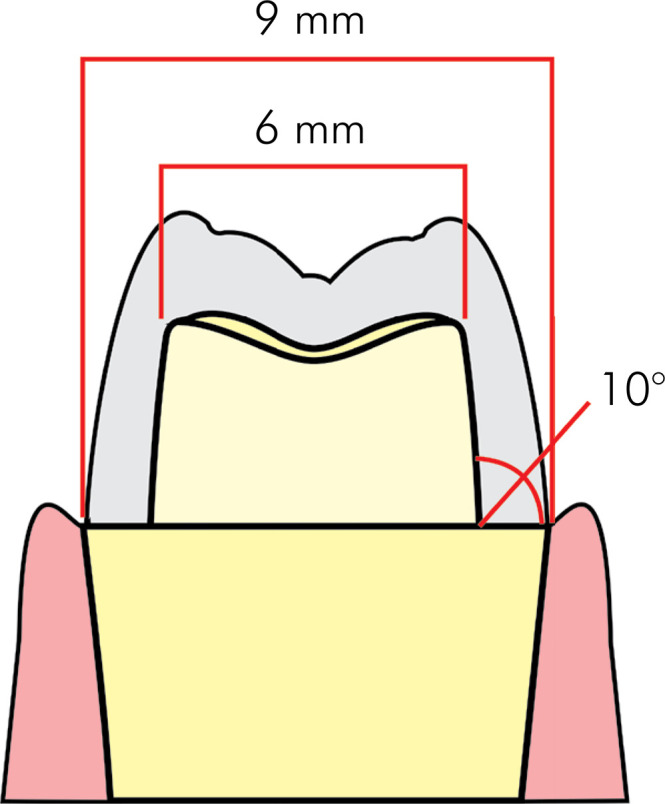
Tooth preparation model.

Thirty human mandibular third molars were collected, selected (Ethics Committee approval 381/06), and fixed in a typodont (Figure 1A). The teeth were measured with a digital caliper (Mitutoyo, Digimatic®, Kawasaki, Japan), which yielded coronal widths varying between 9.5 mm and 10.7 mm. The teeth were randomly divided into three groups (n = 10), and sample size was determined based on previous studies.^9,15^ Diamond burs (1014, 3145, 3098, and 3098F, KG Sorensen, Curitiba, Brazil) with copious air-water spray were used to prepare the all ceramic crowns (Figure 1B), with a rounded shoulder axiogingival angle and depth of wear on the occlusal, buccal and lingual surfaces of 1.5 mm (Figure 2).^32^ Standardized digital impressions of the prepared teeth were generated using a Cerec 3D Omnicam scanner (Sirona Dental Systems GmbH, Bensheim, Alemanha). The crowns were designed with Cerec 3D software (version 4.2.5, Bensheim, Alemanha) (Figure 1C) with different luting space settings: LD40 – 40 μm, LD80 – 80 μm, and LD160 – 160 μm (Figure 1D). The lithium disilicate blocks (IPS e.max, Ivoclar Vivadent AG, Schaan, Liechtenstein) were milled using the Cerec inLab MC XL milling unit (Sirona Dental Systems GmbH) (Figure 1E). The crowns obtained were taken to a specific furnace (Programat P300, Ivoclar Vivadent, Schaan, Liechtenstein) for crystallization, following the programming indicated by the manufacturer.

### Scanning procedures—internal and marginal measurement procedures

Each ceramic crown was fixed by using silicone-based material on each tooth (GC Fit Checker, GC Dental Industrial Corp, Tokyo, Japan) (Figure 1F). To standardize the measurements, each specimen received four radiopaque lines on the cervical portion of the root to determine the buccal, distal, lingual and mesial faces. Each specimen was fixed in the same position on μ-CT (Brucker-Skyscan 1272, Kontich, Belgium) (Figure 1G) to obtain images for measurement of marginal and internal fit. The µ-CT scanning was performed at 100 kV and 100 μA, with a pixel size of 9.4 μm, Filter Cu 0.11 mm, and resolution of 1632 × 1092 pixels. The selected scanning was performed at 1 degree of rotation step at 360 degrees; to diminish artifacts, an average of 2 frames were collected with 20 random movements, resulting in a scanning time of 1 hour per specimen. Images of each specimen were reconstructed (NRecon v, Bruker- Micro-CT, Kontich, Belgium) (Figure 1H), providing axial cross-sections of their inner structure. Data Viewer v.1.4.4 software (SkyScan, Kontich, Belgium) was used to view the images in a 3D profile, and the 3D images of each specimen as well as axial images were analyzed to verify image quality.

For assessing the adaptation of the internal margin, two axiogingival, two axial, and two occlusal sites were measured in the central sagittal and coronal images of the crown, and mean values were obtained for the axiogingival, axial and occlusal sites (Figure 1I).

Thirteen sagittal images were selected for measuring the buccal and lingual marginal fit. Thirteen coronal images were selected for measuring the mesial and distal marginal fit. For each image, vertical fit was measured at 300× magnification using CTAn processing software to evaluate the marginal fit (Version 1.12.0.0, Skyscan). For vertical fit, the measurements were taken from the external crown margin to the most external point of the tooth. The examination of vertical fit at 13 sites in four areas (buccal, lingual, mesial and distal) resulted in 52 measurements per tooth and 520 per group.

### Delivery of irradiance and the emission spectrum of curing units

Before fixation, all crowns were measured. The occlusal, buccal and lingual thickness and a three block of lithium disilicate were reproduced at 1.3 mm, 1.7 mm and 1.8 mm, respectively. Five light-curing protocols were used to verify the mean of the emission spectrum of the curing unit and mean of irradiance delivered using a resin simulator (Blue Light Analytics Inc., Halifax, Canada) in two situations: with 0 mm sensor distance and through the ceramic block of differing thickness: 1.3 mm for occlusal, 1.7 mm for buccal, and 1.8 mm for lingual. The device had a cosine-corrector optical fiber irradiance probe capable of capturing all emitted light, which in turn was guided into a spectral calibrated radiometer. The MARC resin simulator data were transferred to a computer for analysis of emission spectrum peak (in nm) and mean irradiance delivery (in mW/cm^2^) using software (version 3.0.4.0).

### Treatment of the crown surface

The intaglio surface of the ceramic crowns was etched with 5% hydrofluoric acid (Porcelain etchant; Bisco, Schaumburg, USA) for 20 seconds followed by a water spray for 10 seconds and air drying for 20 seconds. One coat of silane (RelyX Ceramic Primer; 3M ESPE, St. Paul, USA), which was applied for 20 seconds using a microbrush, was then air-dried for 10 seconds in room-temperature air.

### Strain gauge fixation

Strain gauges were bonded to the buccal and distal ceramic crown surfaces (n = 10) with cyanoacrylate adhesive (Super Bonder; Loctite, Düsseldorf, Germany) and were connected to a data acquisition device (ADS0500IP; Lynx Tecnologia Eletrônica). The buccal strain gauge (PA-06-060CC-350L; Excel Sensores, São Paulo, Brazil) had an internal electrical resistance of 350 Ω, a gauge factor of 2.07, and a grid size of 21.02 mm^2^. The distal strain gauge had an internal electrical resistance of 120 Ω, a gauge factor of 2.137, and a grid size of 1.00 mm^2^. The gauge factor is a proportional constant between electrical resistance variation and strain. The dimension of the face defined the size of the strain gauges, and the flat area was selected to fix the strain gauge. In addition, two strain gauges were fixed to another intact tooth to compensate for dimensional deviations due to temperature effects such as in a passive specimen.

### Crown luting procedure and deformation analyses

A self-adhesive dual cured resin cement (Rely X U200; 3M ESPE, St. Paul, USA) was selected for the crown luting procedure. The resin cement was mixed and applied to the inner surface of the ceramic crowns, which were inserted onto the tooth preparation and remained seated under finger pressure. Ceramic crown deformation was measured during the crown stabilization and self-curing polymerization for 5 minutes. Excess cement was removed with a dry microbrush, and resin cements were light-activated using a monowave light-curing unit (Radii-Cal unit; SDI, Victoria, Australia) operated at 1200 mW/cm^2^ for 40 seconds on each surface: buccal, lingual and occlusal, resulting in 120 seconds, totalizing 144 J. Ceramic crown deformation data were obtained with the strain gauges with data analysis software (AqDados 7.02 and AqAnalisys; Lynx, São Carlos, Brazil). The strain values were recorded at 4 Hz during fixation and during light activation and sustained for 5 minutes after the polymerization process.

### Statistical analysis

Internal adaptation, marginal fit, irradiance emitted by the light-curing device, and ceramic crown deformation data were tested for normal distribution (Shapiro-Wilk) and variance equality (Levene test). Data were analyzed by one-way analysis of variance (ANOVA) followed by Tukey’s post-hoc test for pair-wise comparisons. All analyses were performed using SigmaPlot statistical software (version 12.0; Systat Software Inc., San Jose, USA) with a significance level of α = 0.05.

## Results

**Figure 3:**
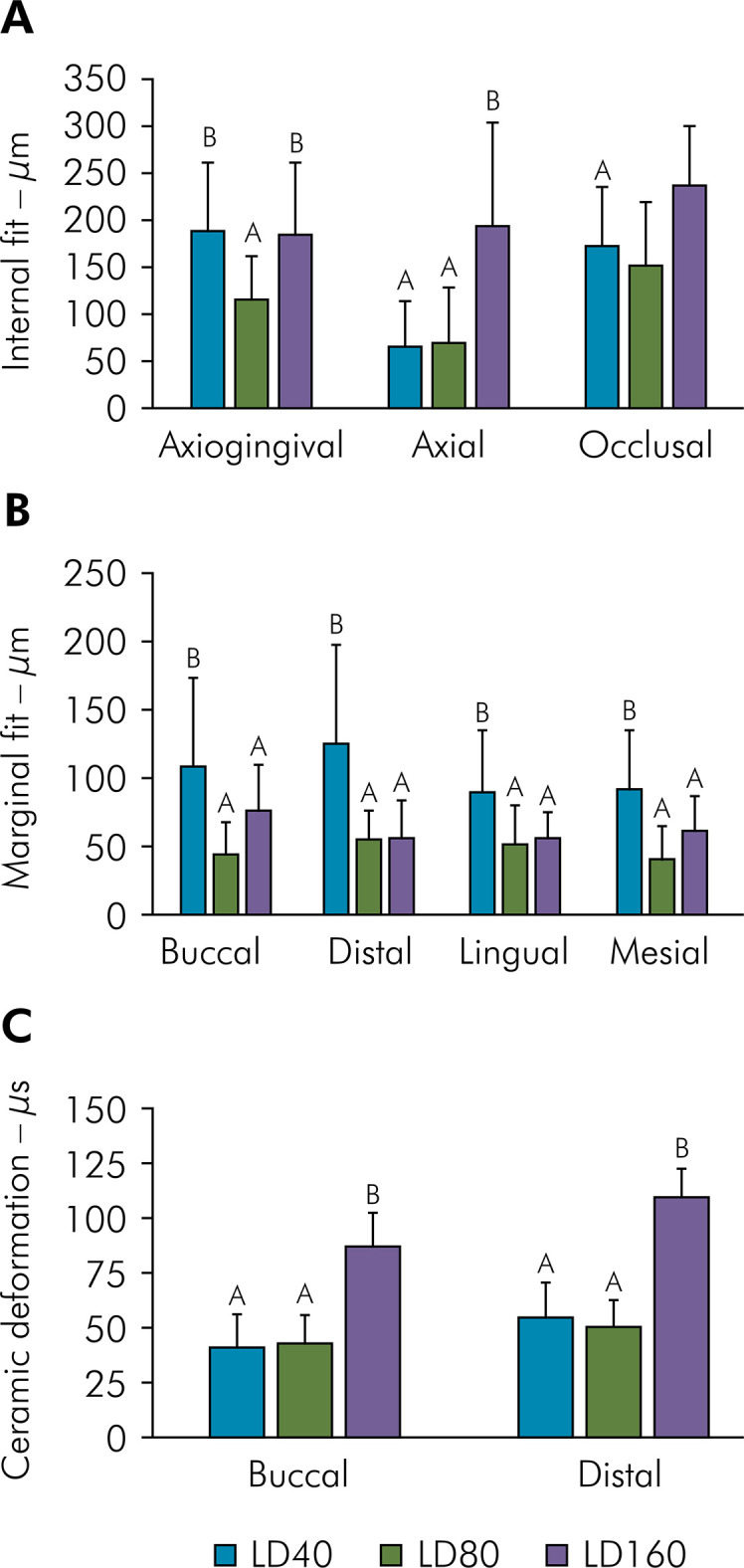
(A) Internal fit of lithium disilicate crowns with different luting space settings (in μm). (B) Marginal fit of lithium disilicate crowns with different cement thickness (in μm). (C) Deformation of lithium disilicate ceramic crowns at buccal and distal sites, in terms of microstrain (μS) cementation shrinkage polymerization (μS).

Figure 3A presents mean and standard deviation data for internal gap, measured considering axiogingival, axial and occlusal walls (Table 1). The internal gap differed significantly among the axiogingival (df = 2, F = 7.4, p = 0.001), axial (df = 2, F = 17.3, p < 0.001), and occlusal walls (df = 2, F = 4.9, p = 0.01), as assessed by ANOVA. Tukey’s test demonstrated that LD160 resulted in a significantly larger occlusal and axial internal gap than LD40 or LD80 (p < 0.001). For axiogingival fit, LD80 demonstrated significantly smaller values than LD40 and LD160 (p = 0.003, p = 0.006).

**Table 1:** Means and standard deviation (±SD) of internal and marginal fit.

Groups	Internal fit (µm)			Marginal fit (µm)			
	Axiogengival	Axial	Oclusal	Buccal	Distal	Lingual	Mesial
LD40	187.9 ± 70.8 ^B^	65.5 ± 47.3^A^	171.6 ± 61.1^A^	110.5 ± 50.6^b^	115.6 ± 60.2^b^	86.2 ± 49.2^b^	92.9 ± 24.9^b^
LD80	115.4 ± 44.6^A^	68.6 ± 35.9^A^	150.8 ± 73.2^A^	46.2 ± 17.0^a^	58.2 ± 22.9^a^	50.6 ± 26.9^a^	43.4 ± 23.8^a^
LD160	183.4 ± 75.5^B^	192.7 ± 73.1^B^	236.6 ± 61.02^B^	66.7 ± 33.1^a^	56.5 ± 27.0^a^	52.0 ± 28.5^a^	56.6 ± 27.6^a^

Values with the same superscript letter are not significantly different (p < 0.05).

Figure 3B presents mean and standard deviation data for vertical gap, recorded by region and luting space (Table 1). Vertical gap differed significantly among all regions: buccal (df = 2, F = 4.2, p = 0 .025), lingual (df = 2, F = 4, p = 0.029), medial (df = 2, F = 6.1, p = 0.006), and distal (df = 2, F = 7.5, p = 0.002) (ANOVA). Tukey’s test demonstrated that LD40 resulted in a significantly larger marginal gap than LD80 or LD160, irrespective of region.

Figure 3C presents mean and standard deviation data for ceramic crown deformation, measured with the buccal and distal strain gauges (Table 2). Ceramic deformation differed significantly for the buccal (df = 2, F = 32.0, p < 0.001) and distal (df = 2, F = 56.8, p < 0.001) sites (ANOVA). Tukey’s test demonstrated that LD160 resulted in significantly greater deformation than LD40 or LD80, irrespective of region (p < 0.001).

**Table 2:** Deformation of the buccal and distal surfaces of crowns obtained by CAD/CAM with different cementation spaces.

Groups	Ceramic deformation (µS)	
	Buccal	Distal
LD40	40,9 ± 15,2^A^	54,6 ± 15,8^a^
LD80	42,8 ± 12,9^A^	50,4 ± 12,0^a^
LD160	86,9 ± 15,2^B^	109,1 ± 12,9^b^

Values with the same superscript letter are not significantly different (p < 0.05).

**Figure 4:**
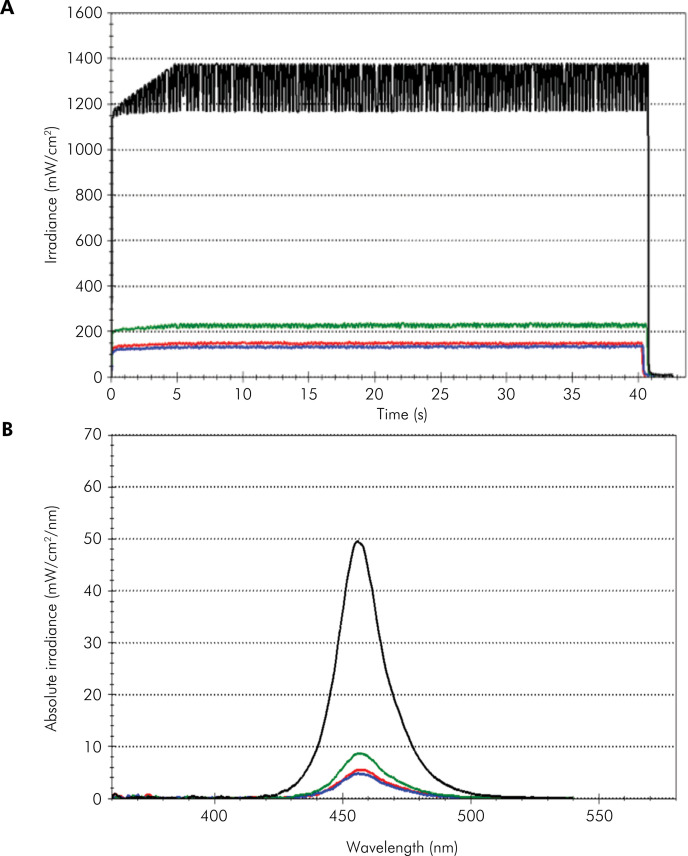
(A) Light spectrum emitted by the curing unit (Radii-cal) through ceramics of varying thickness (in nm). (B) Irradiance delivery (in mW/cm2) by the curing unit through ceramics of varying thickness. Black lines, without ceramic; green lines, occlusal ceramic thickness; red lines, buccal ceramic thickness; blue lines, lingual ceramic thickness.

Figures 4(A and 4B shows the irradiance (also refer to Table 3) for two situations: 0 mm from the sensor or through various thicknesses of ceramic block and then to the sensor. The irradiance delivered by the curing unit was reduced significantly upon interposition of the ceramic block to the sensor (df = 3, F = 133331.805, p < 0.001). The light spectrum presented a wavelength between 420 nm and 500 nm, and the peak wavelength was similar for all tested groups (df = 3, F = 0.591, p < 0.630).

**Table 3:** Means and standard deviation (±SD) of irradiance.

Ceramic	Irradiance (mW/cm^2^)
0 mm	1243.5 ± 4.9
1.3 mm	224.8 ± 1.9
1.7 mm	171.9 ± 1.5
1.8 mm	162.4 ± 1.4

Values with the same superscript letter are not significantly different (p < 0.05).

## Discussion

The null hypothesis of this study was rejected. The luting space settings influenced the internal fit, marginal fit, and ceramic deformation as a result of polymerization shrinkage of the resin cement used for cementing the CAD/CAM lithium disilicate ceramic crowns. The irradiance delivered to the cement decreased with increasing thickness of the ceramic, although all spectra were similar.

The internal gap was measured for each crown at carefully selected sites, as determined by μ-CT. A reduced internal space of crowns optimizes the cementation process.^2-4^ However, it is clear that when the smallest luting space is selected, internal contacts can occur within the axial walls of the preparation, thus influencing the crown seating and the final marginal fit.^5,12,34^ Intermediary values of luting space settings (80 μm) yielded the best results for internal fit based on axiogingival measurements. The LD40 and LD80 groups had similar internal fit data for both axial and occlusal measurements. However, the crowns fabricated with LD160 resulted in significantly higher values for axial and occlusal internal fit. Considering the occlusal luting space settings, all groups yielded values > 100 μm. This situation could generate critical stress and possible crown failure.^3,4^ Moreover, our results demonstrate that precise manufacturing is not possible for reproducing luting space settings, as determined with software, to yield a specific internal fit of crowns.

Regarding marginal fit, reduced luting space settings promote higher misfit values. Previous studies yielded similar results.^1,2,6,37^ Inner contacts between the crown and preparation increase the margin gap because the crown does not seat completely, resulting in soft-tissue injury and the potential for developing secondary caries.^1,2,5,6,16^ In the present study, crowns milled with a luting space setting of 80 μm or 160 μm yielded better results than 40 μm. This may be attributable to the fact that, in situations of larger inner space, there is no internal contact to allow for better seating. In this situation, mean values were < 75 μm, which is clinically acceptable.^9,15,38^

On the other hand, if luting space exceeds 80 μm, the polymerization process could be influenced.^2,5^ This could be observed when the luting space was set at 160 μm. Crown deformation was analyzed at two sites, namely buccal and distal, using two strain gauges, confirming the finite element results of other studies.^3,4,10^ Crowns produced with 160 μm luting space presented higher deformation compared with the LD40 and LD80 groups. This suggests that LD160 can increase the stress on the cement line, generating ceramic microcracks and bonding failure of the crown, which may compromise the longevity of ceramic restorations. Shrinkage stress is a serious concern, as has been demonstrated clinically by the high incidence of loss of marginal integrity. Indeed, studies have shown that a large cement margin increases the deformation of the ceramic crown surface.^3,4,10^ Finite element analyses have shown little effect of tensile stress when occlusal cement thickness of ceramic crows is thinner.^3,4,20^ A cement-layer thickness of > 100 μm on the occlusal region of the internal surface of the crown can result in a critical level of stress on the axial-occlusal line.^3,4^

The shrinkage process is dependent on the extent of the resin cement polymerization, which may be influenced by the incident irradiance emitted by the light-curing unit.^22,25,26,28^ Thus, the current study characterized the irradiance delivered by the curing unit used in the experiment and also the effect of ceramic thickness in the occlusal, buccal and lingual regions.^29^ The polymerization process could differ at different sites, and shrinkage was measured as a function of the standard time of polymerization. As observed, the higher the ceramic thickness, the lower irradiance reaching the resin cement lays. This aspect clearly shows that the light-activation time defined for resin cement should be considered as well as the irradiance potential of the light-curing unit. The Radii-cal device we used delivered high-level irradiance as measured directly at the sensor; however, when the ceramics were placed over the sensor, the irradiance was reduced significantly. It is important to note that another type of curing unit might deliver a different irradiance and spectrum, which might affect the amount of polymerization shrinkage.

The results of the present study suggest that selecting a setting of 80 μm for luting space would favor the marginal and internal fit, thereby promoting clinically acceptable seating and minimizing crown deformation that can result from shrinkage stress of the resin cement layer. However, future studies could use values lower than 80 μm but higher than 40 μm to ascertain the best luting space value and, thereafter, crowns could be subjected to cyclic loading or fracture and adhesive strength tests while varying luting space. Further, other curing units could be evaluated, and polymerization time could be measured as to different ceramic thickness to determine the influence on crown deformation.

Finally, the present study has limitations, as it could not replicate all the nuances of oral results that may influence these findings. However, internal adjustments were implemented at internal sites where there was no cementing space.

## Conclusion

Within the limitations of this in vitro study, we conclude the following: (a) The optimal luting space was 80 μm, which reduced the internal and marginal gaps and minimized both resin cement stress and ceramic deformation in lithium disilicate CAD/CAM crowns; (b) reducing the luting space settings may result in internal contacts with the axial walls, thus preventing proper seating of ceramic crowns; and (3) increasing the luting space settings increases resin cement shrinkage stress, leading to greater deformation of ceramic crowns.

## Data Availability

Data is available on demand from the reviewers.
